# Extracellular vesicles from endothelial progenitor cells promote thyroid follicle formation

**DOI:** 10.1080/20013078.2018.1487250

**Published:** 2018-06-28

**Authors:** Jonathan Degosserie, Charlotte Heymans, Catherine Spourquet, Mathias Halbout, Ludovic D’Auria, Patrick Van Der Smissen, Didier Vertommen, Pierre J. Courtoy, Donatienne Tyteca, Christophe E. Pierreux

**Affiliations:** a CELL Unit, de Duve Institute, Université Catholique de Louvain, Brussels, Belgium; b BCHM Unit, de Duve Institute, Université Catholique de Louvain, Brussels, Belgium; c PICT plateform, de Duve Institute, Université Catholique de Louvain, Brussels, Belgium; d Neurochemistry group, CEMO division, Institute of Neuroscience, Université Catholique de Louvain, Brussels, Belgium; e PHOS Unit, de Duve Institute, Université Catholique de Louvain, Brussels, Belgium

**Keywords:** Thyroid, folliculogenesis, endothelial cells, extracellular vesicles, laminins

## Abstract

Organogenesis is a complex and dynamic process requiring reciprocal communication between different cell types. In the thyroid, thyrocyte progenitors secrete the angiocrine factor, VEGFA, to recruit endothelial cells. In return, endothelial cells promote thyrocyte organisation into spherical follicular structures, which are responsible for thyroid hormone synthesis and storage. Medium conditioned by endothelial progenitor cells (EPCs) can promote follicle formation and lumen expansion (i.e. folliculogenesis) in an *ex vivo* culture system of thyroid lobes. Here, we postulated that endothelial cells instruct thyrocyte progenitors by producing extracellular vesicles (EVs). We found that medium conditioned by EPCs contain EVs with exosomal characteristics and that these vesicles can be incorporated into thyrocyte progenitors. By mass spectrometry, laminin peptides were abundantly identified in the EV preparations, probably co-sedimenting with EVs. Laminin-α1 silencing in EPC abrogated the folliculogenic effect of EVs. However, density gradient separation of EVs from laminins revealed that both EV-rich and laminin-rich fractions exhibited folliculogenic activity. In conclusion, we suggest that endothelial cells can produce EVs favouring thyrocyte organisation into follicles and lumen expansion, a mechanism promoted by laminin-α1.

## Introduction

The thyroid is an endocrine gland composed of three main cell types: thyrocytes, C cells and endothelial cells. Thyrocytes are polarised epithelial cells organised as monolayers into follicles enclosing packed iodothyroglobulin in the colloid, and responsible for the production of thyroglobulin-derived hormones, T3 and T4. C cells are epithelial cells, closely associated with follicles, which produce calcitonin. A dense network of endothelial cells surrounds each thyroid follicle. The close juxtaposition between follicles and blood capillaries is essential for bidirectional exchange supporting thyroid function. Iodide is captured from bloodstream into thyrocytes, and incorporated into thyroglobulin as T3 and T4 hormonogenic peptides. When T3 and T4 blood levels are low, thyrocytes capture iodothyroglobulin and release by proteolysis T3 and T4 hormones that are excreted into the blood flow. The functional couple between follicles and blood capillaries, often called the angio-follicular unit, forms during embryonic development [1,2].

During embryogenesis, thyrocyte progenitors derive from the endoderm upon expression of a combination of four transcription factors (Nkx2.1, Pax8, Foxe1 and Hhex), and first form a mass of non-polarised cells. The bud of thyrocyte progenitors migrates ventrally and then elongates laterally to form two lobes, on each side of the trachea, linked by the isthmus. By the end of gestation, thyroid progenitors differentiate and organise into a multitude of follicles associated with their blood capillaries [3]. We have demonstrated, by genetic inactivation of *Vegfa* in embryonic mouse thyroid, that endothelial cells are essential for the organisation of thyrocyte progenitors in follicles [4]. Using an *ex vivo* culture system of thyroid lobes, we further showed that endothelial progenitor cells (EPCs) act on thyroid development independently of the blood flow, by secreting one or several instructive factors that promote the formation of spheroid structures delineated by a monolayer of differentiated thyrocytes and their luminal expansion (hereafter referred to as “folliculogenesis”). More recently, we reported that folliculogenesis is also controlled by BMP ligands. Indeed, thyroid-specific deletion of *Smad1* and *Smad5* display defective follicular organisation and disrupted basement membrane assembly [5]. Furthermore, unpublished data from our group revealed that the folliculogenic activity could be retrieved not only from total medium conditioned by EPCs, but also from a crude sedimentation of this medium. Whether this sedimentable activity corresponds to extracellular vesicles (EVs), and whether EVs could control thyroid follicle formation, is yet unknown.

EVs are small membrane-bound vesicles produced by almost every cell type and involved in intercellular communications [6]. EVs were first described in the early 80’s as “garbage bags” used by maturing reticulocytes to dispose of transferrin receptor [7,8]. However, in the last decade, EVs have been shown to carry proteins and RNAs, and their potential as vehicles for intercellular communication was revealed [9,10]. EVs can be divided into three main populations: (i) large-size EVs include apoptotic bodies and oncosomes; (ii) microvesicles (MVs) of medium size (~100–1000 nm) are produced by plasma membrane budding; and (iii) exosomes are small-size EVs (<150 nm) produced by the endosomal machinery [11,12]. Communication through EVs has been shown in a large variety of physiological and pathological processes [11,13]. Recently, exosomes isolated from developing submandibular glands have been shown to control expansion of epithelial progenitors during organogenesis [14]. Yet, the role of EVs in embryonic development is poorly known.

The goal of the present study was to examine whether endothelial cells can stimulate folliculogenesis via the release of EVs. We show that EPCs produce EVs with exosomal characteristics that stimulate folliculogenesis in thyroid lobes cultured *ex vivo*.

## Materials and methods

We have submitted all relevant data of our experiments to the EV-TRACK knowledgebase (EV-TRACK ID: EV170013) [15].

### Animals

All mice were of CD1 strain. Mice were raised and treated according to the principles of laboratory animal care of the University Animal Welfare Committee. Females were mated and the day of the vaginal plug was considered as embryonic (E) day 0.5. At the indicated day of gestation, mice were sacrificed by cervical dislocation.

### Dissection and culture of thyroid lobes

Thyroid lobes micro-dissected from E14.5 mouse embryos were cultured as described for 3 days [16]. Briefly, lobes were placed on microporous filters (Millipore) at the air:medium interface in M199 medium (Gibco) supplemented with 10% foetal bovine serum (FBS, Sigma), 100-U/mL penicillin, 100-µg/mL streptomycin, 0.25-µg/mL fungizone and 2-mM L-glutamine, supplemented by ~6 10^8^ EVs (corresponding to 1.3-µg protein) for 10^4^ cells or not (control condition). Supernatant from ultracentrifuged medium (EV-low EPC-CM) was concentrated 10-fold on Amicon Ultra-4, with a 50-kDa nominal cut-off (as in [4]), before incubation with the thyroid lobes. To ensure optimal contact with explants, 10 µL from the culture medium (control, EPC-EVs, sh-EVs, GipZ-EVs, EV-rich, laminin-rich or EV-low medium) was added on top of the filter, three times per day.

### Immunolabeling on sections

Cultured thyroid explants were fixed in 4% formaldehyde for 1 h and embedded in gelatin. Immunofluorescence was performed on 7-µm sections as described [17] using anti E-cadherin (1:1,000, BD Bioscience) and anti-ezrin (1:200, Thermo Fisher Scientific) primary antibodies, followed by secondary antibodies coupled to Alexa-488 or Alexa-568 (Thermo Fisher Scientific). Alternatively, fixed explants were immunolabelled as whole mount [4]. Fluorescence was observed with a Zeiss Cell Observer Spinning Disk microscope (objective 40×, oil immersion).

Quantification of ezrin^+^ follicles was performed at least on five sections spanning each lobe using a Zeiss Observer Z1 inverted fluorescence microscope. Open follicles were counted manually when lumen was > 10 µm. Values were normalised to untreated explants, considered as unity. Per condition, a minimum of six lobes, from three independent experiments, was used for quantifications.

### Cell culture

Embryonic EPCs [18] were cultured on gelatin-coated dishes in DMEM (Westburg) supplemented with 20% FBS (Sigma), 100-U/mL penicillin, 100-µg/mL streptomycin and 100-µM non-essential amino acids. Cells were trypsinised using Tryple Express (Invitrogen). Conditioned medium (CM) was prepared by incubating 80% confluent EPC in M199 without serum for 24 h (7 mL/78 cm^2^ dish). Transduction of cells was performed with pGIPZ lentiviral vectors as described and validated in Villacorte *et al*. [5].

For primary cultures, E14.5 thyroid lobes were micro-dissected and washed in DPBS (Gibco) containing 0.2-mM EDTA. Thyroid lobes were gently dissociated in Accutase**®** (ThermoFisher Scientific) for 30 min at 37°C and the enzymatic reaction was stopped by adding M199 containing 10% FBS. Dissociated cells were counted and seeded in Ibidi**®** chambers suited for microscopy (Ibidi) at 50,000 cells per well (100 mm^2^) in M199 containing 10% FBS. After overnight incubation at 37°C, dead or non-attached cells were discarded by medium replacement and culture was continued for an additional 3 h before analysis.

### EV isolation

EPC-CM was collected after 24 h and submitted to differential ultracentrifugation: 1,000× *g* for 10 min (GR 4.11, Jouan), 20,000× *g* for 15 min (referred to as 20k pellet) in a Ti50 rotor (Beckman), and 150,000× *g* for 90 min (first 150k pellet) in a Ti80 rotor (Beckman). The first 150k pellet was washed once by suspension in PBS and resedimentation at 150,000× *g* for 90 min. This material is hereafter referred to as “150k pellet” and was finally collected in PBS or in appropriate medium, depending on further application.

### Density gradient

Alternatively, the 150k pellets were further purified by floatation in iodixanol gradient as described [19]. Briefly, 1.3 mL of sample was mixed with 1.3 mL of 60% iodixanol stock solution (Axis-Shield) to obtain a 30% iodixanol layer, placed at the bottom of the tube. Then, 1.2 mL of 20% and 1.2 mL of 10% iodixanol, diluted from the stock solution, were sequentially added on the top of the 30% layer to obtain three layers in the gradient with a final volume of 5 mL. Gradients were centrifuged at 100,000× *g* for 16 h in a swinging bucket rotor (SW55 rotor, Beckman), and eight fractions (denoted F1 to F8 from top to bottom) of 625 µL were collected by aspiration from top, diluted in PBS pelleted again at 150,000× *g* for 90 min in a Ti80 rotor as above, and suspended in the appropriate medium. To test biological activity, we used 2/3 of EV-rich (F1–4) as buoyant fraction, 2/3 of laminin-rich (F5–8) as dense fraction or 1/3 of F1-4 mixed with 1/3 of F5-8 as reconstituted 150k pellet. Density of each fraction was calculated, using an equilibrating gradient, as mass/volume (g/cm^3^) by reference to water (1g/cm^3^).

### Nanoparticle tracking analysis

EVs were counted in samples before and after separation by floatation in iodixanol gradient by the Zetaview (Particle Metrix, GmbH), which captures Brownian motion through a laser scattering microscope combined with a video camera to obtain size distribution (50–1000 nm) and concentration. DBPS (without calcium and magnesium, ThermoFisher) pre-filtered on 100-nm nylon membrane or M199 medium (Gibco) was used to dilute samples. Samples were diluted 1:10–1:500 to reach 50–200 particle/frame corresponding to ~2 10^7^–1 10^8^ particles/mL. Sensitivity was set to 72.2 and camera shutter to 70 in order to detect less than 3 particles/frame as background when DBPS alone was injected. Measurements were performed with medium resolution for two cycles at 11 different positions (or frames) of the cell chamber containing the sample; measurements were reproduced 2–3 times. The Zetaview software was used to generate results in text files. Graphs for particle size and distribution were designed with Prism (GraphPad software).

### EV fluorescent labelling

To label EPC-EVs, the suspended 150k pellet was incubated with the membrane dye PKH67 (Sigma) according to manufacturer’s instructions. Free dye was then eliminated by the density gradient. The four first fractions, containing labelled EVs, were pooled, washed in PBS and collected by ultracentrifugation at 150,000× *g* for 90 min.

### EVs incorporation and immunofluorescence on cells

The equivalent of 10-µg protein of PKH67-stained EVs was incubated with a primary culture of embryonic thyroid cells. After 3 h, cells were rinsed extensively with PBS to remove remaining particles, fixed with 4% formaldehyde pH 7.4 for 15 min, and then washed again extensively. Cells were further permeabilised with 0.05% saponin pH 7.4 for 15 min and incubated for 30 min in blocking solution (1% BSA; 0.1% lysine; 0.01% saponin in PBS). Permeabilised cells were then incubated with anti-CD63 (1:150, Biolegend) primary antibody, diluted in blocking solution for 1 h, washed with blocking solution, incubated with secondary antibody coupled with Alexa-568 (1:1,000, Invitrogen) together with Hoechst 33258 as nuclear dye (1:1,000, Sigma) in blocking solution for 1 h. After final wash in blocking solution, cells were rinsed three times with PBS, post-fixed in 4% formaldehyde for 5 min and mounted in aqueous mounting medium (Dako). Preparations were observed with a Zeiss Cell Observer Spinning Disk microscope (objective 100×, oil immersion).

### Western blotting

For western blotting, 150k pellets were lysed in RIPA buffer (150-mM NaCl; 1% Triton X-100; 0.5% sodium deoxycholate; 0.1% SDS; 50-mM Tris pH 8.0; 1× protease inhibitor cocktail (Roche)). Protein concentration was measured using bicinchoninic acid. Loads normalised to 4-μg protein were resolved on 10% homemade polyacrylamide gels or 4–15% pre-casted polyacrylamide gels (Biorad), then transferred onto PVDF membrane and processed as described [20]. The following primary antibodies were used: anti-calnexin (1:10,000, ADI-SPA-860D Enzo Life Science), anti-CD63 (1:300, 143902 Biolegend; in non-reducing environment), anti-CD9 (1:300, EPR2949 Abcam), anti-flotilin-1 (1:200, 610821 BD Transduction) and anti-laminin-α1 (1:1,000, kind gift from T. Sasaki). Immuno-reactive bands were detected using chemiluminescence (Super Signal Chemiluminescent Substrate; Thermo Scientific) and images were acquired using Fusion Solo S (Vilber Lourmat).

### Scanning electron microscopy

In addition, 150k pellets were allowed to adhere onto poly-L-lysine-coated coverslips then fixed in 1% glutaraldehyde, washed and post-fixed with 1% OsO_4_. Samples were dehydrated in ethanol, critical point-dried and coated with a 10-nm gold film. Specimens were observed with CM12 electron microscope (Philips, Netherlands) at 80 kV with the use of a secondary electron detector, as described [21].

### Mass spectrometry

Moreover, 150k pellets were suspended in 50 µL of 50-mM NH_4_HCO_3_ pH 8.0, reduced and alkylated, and incubated overnight at 37°C with trypsin and 0.2% Rapigest® for enzymatic digestion. Detergent was hydrolysed with HCl and sedimented by centrifugation. Peptides were collected from the supernatant and analysed by liquid chromatographic tandem mass spectrometry (LC–MS/MS). The LC–MS/MS system consisted of an LTQ XL IonTrap mass spectrometer (ThermoScientific, San José, CA, USA) equipped with a microflow ESI source and interfaced to an LCPackings Ultimate Plus Dual gradient pump, Switchos column switching device, and Famos Autosampler (Dionex, Amsterdam, Netherlands). Two RP peptide traps C18 Pepmap 100 Dionex (0.30 mm × 5 mm) were used in parallel with two analytical BioBasic-C18 columns from ThermoScientific (0.18 mm × 150 mm). Samples (6.5 μL) were injected and desalted on the peptide trap equilibrated with solvent A (3.5% ACN v/v, 0.1% TFA in water) at a flow rate of 30 μL/min. After valve switching, peptides were eluted in backflush mode from the trap onto the analytical column equilibrated in solvent B (5% ACN v/v, 0.05% v/v formic acid in water) and separated using a 100-min gradient from 0 to 70% solvent C (80% ACN v/v, 0.05% formic acid in water) at a flow rate of 1.5 μL/min. Peak lists were generated and searched using the bioinformatic software Proteome Discoverer 1.4.1 (Thermofischer Scientific). In details, peak lists were generated using extract-msn (ThermofischerScientific) within Proteome Discoverer 1.4.1. From raw files, MS/MS spectra were exported with the following settings: peptide mass range 350–5000 Da, minimal total ion intensity 500. The resulting peak lists were searched using SequestHT against a target-decoy mouse or human protein database obtained from Uniprot. The following parameters were used: trypsin was selected with proteolytic cleavage only after arginine and lysine, number of internal cleavage sites was set to 1, mass tolerance for precursors and fragment ions was 1.0 Da, considered dynamic modifications were + 15.99 Da for oxidised methionine and +57.00 for carbamidomethyl cysteine. Peptide matches were filtered using the *q*-value and Posterior Error Probability calculated by the Percolator algorithm ensuring an estimated false positive rate below 5%. The filtered Sequest HT output files for each peptide were grouped according to the protein from which they were derived and their individual number of peptide spectral matches was taken as an indicator of protein abundance. Finally, identified proteins were manually validated.

### Statistical analysis

Statistical analyses were performed with Prism software (GraphPad) using Kruskal–Wallis test with Dunn post-test for ezrin^+^ follicles quantification. Student’s *t*-test was performed for western blot quantification. Data are presented as means ±SD.

## Results

### Endothelial progenitor cells produce extracellular vesicles with exosomal characteristics

**Figure 1.**
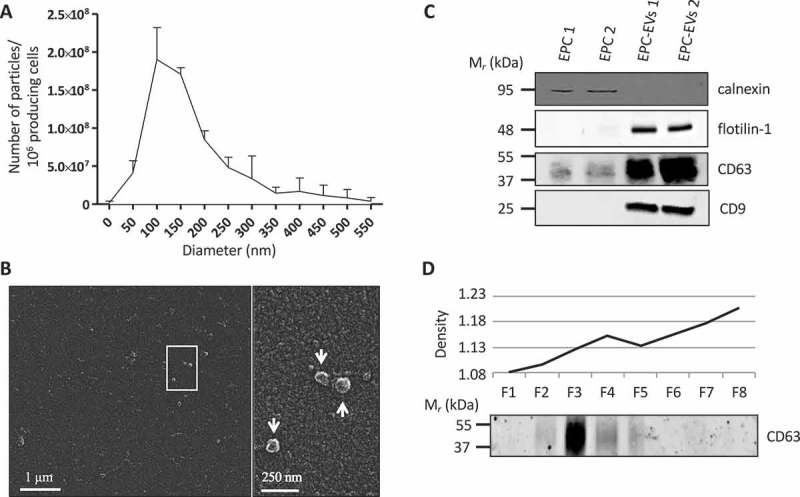
EVs isolated from EPC-CM display exosomal properties. (A) Quantification and size distribution of EVs obtained by Nanoparticle Tracking analysis using a Zetaview (Particle Metrix). Results are presented as the number of particles per 10^6^ producing cells. (B) Representative scanning electron microscopy image of the 150k pellet obtained from EPC-CM. Enlarged view of the boxed area is shown at the right panel. EVs are indicated by arrows. (C) Western blot analysis of calnexin (ER marker) and flotilin-1, CD63 and CD9 (EVs markers) in two EPCs and two EPC-EV lysates. (D) Western blot analysis of CD63 on eight fractions (F1–F8) resolved by floatation in iodixanol density gradient. Density of the eight fractions is shown in the upper graph.

We have previously shown that endothelial cells are essential to promote thyroid folliculogenesis, and that medium conditioned (CM) by EPCs can promote folliculogenesis in thyroid explants [4]. Furthermore, crude high-speed ultracentrifugation of the CM revealed that the sedimentable material was endowed with folliculogenic activity. Here, we first tested whether this activity could be ascribed to EV generated by EPCs. To this aim, medium conditioned by EPC was submitted to differential ultracentrifugation [22,23]. We focused on the 150k pellet, which was analysed with a nanotracking device and by scanning electron microscopy. For size distribution in the hydrated state, the 150k pellet was analysed using a particle analyser, which defined a main population with a mean size around 100 and 150 nm and quantified the amount of vesicles (Figure 1(A)). In this range of size, approximately 3.6 10^8^ particles were obtained per 10^6^ producing cells. The electron microscope revealed round monomorphous objects of 100 nm as approximate size in the dried state, consistent with vesicles (Figure 1(B)). Western blotting characterisation of the 150k pellet in comparison with total lysate from the parental EPC cell line showed enrichment in CD63, flotilin-1 and CD9, three well-known EV markers (Figure 1(C)). Conversely, calnexin, a protein of the endoplasmic reticulum, was not detected in the 150k pellet, arguing against significant contamination by intracellular membrane compartments. These biochemical data were confirmed by mass spectrometry analysis (see below, green lines in Table 1). Finally, equilibration of the 150k pellet on iodixanol density gradient revealed that CD63^+^ vesicles floated at a low buoyant density between 1.11 and 1.13 (Figure 1(D)). Altogether, these data indicated that EPC produce EVs with exosomal characteristics, as proposed by the International Society for Extracellular Vesicles [24].

**Table 1.** Comparative mass spectrometry analysis of the 150k pellet isolated from EPC-CM and HUVEC-CM reveal abundant laminin-α1 peptides. Upper 20 lanes (red and white) correspond to proteins identified in the EPC 150k pellet with the highest score in the mass spectrometry analysis, and for which no orthologous protein was identified in the HUVEC 150k pellet. Lower lanes (green) correspond to four known EV markers identified in the EPC 150k pellet. Syntenin-1 and CD9 were also identified in the HUVEC 150k pellet.

Accession	Description	Score	Coverage	No. of peptides	MW [kDa]
P19137	Laminin subunit alpha-1 [LAMA1_MOUSE]	1033.99	33.72	79	338
O88322	Nidogen-2 [NID2_MOUSE]	105.16	24.52	21	153.8
P21956-2	Isoform 2 of Lactadherin [MFGM_MOUSE]	57.58	33.33	10	47.1
P80314	T-complex protein 1 subunit beta [TCPB_MOUSE]	33.07	25.61	8	57.4
Q60675	Laminin subunit alpha-2 [LAMA2_MOUSE]	32.21	0.8	2	342.5
H7BX99	Prothrombin [H7BX99_MOUSE]	29.76	6.65	5	70.2
P58022-2	Isoform 2 of Lysyl oxidase homolog 2 [LOXL2_MOUSE]	29.51	12.65	6	81.1
Q9Z1R9	MCG124046 [Q9Z1R9_MOUSE]	27.29	8.13	1	26.1
L7N202	Uncharacterised protein [L7N202_MOUSE]	24.3	32.33	8	29.9
P68033	Actin, alpha cardiac muscle 1 [ACTC_MOUSE]	23.43	16.45	5	42
P68368	Tubulin alpha-4A chain [TBA4A_MOUSE]	23.18	29.24	8	49.9
E9Q5F6	Polyubiquitin-C (Fragment) [E9Q5F6_MOUSE]	22.98	32.84	2	22.6
F6YTZ4	Uncharacterised protein [F6YTZ4_MOUSE]	20.18	24.09	5	30.1
P10852	4F2 cell-surface antigen heavy chain [4F2_MOUSE]	19.56	16.54	6	58.3
G3XA35	MCG116562, isoform CRA_a [G3XA35_MOUSE]	15.76	2.13	3	262.6
P63038	60 kDa heat shock protein, mitochondrial [CH60_MOUSE]	15.46	8.55	3	60.9
Q99JI6	Ras-related protein Rap-1b [RAP1B_MOUSE]	14.57	20.65	3	20.8
Q8VDN2	Sodium/potassium-transporting ATPase subunit alpha-1 [AT1A1_MOUSE]	12.41	4.3	3	112.9
F6YVP7	Uncharacterised protein [F6YVP7_MOUSE]	12.03	25.66	5	17.7
Q99PV0	Pre-mRNA-processing-splicing factor 8 [PRP8_MOUSE]	11.8	2.06	2	273.4
A2AKJ9	Syntenin-1 [A2AKJ9_MOUSE]	11.53	21.11	2	21.5
P40240	CD9 antigen [CD9_MOUSE]	5.68	11.06	1	25.2
P35762	CD81 antigen [CD81_MOUSE]	5.2	8.47	1	25.8
P41731	CD63 antigen [CD63_MOUSE]	2.67	4.62	1	25.7

### Embryonic thyroid epithelial cells incorporate EPC-EVs

**Figure 2.**
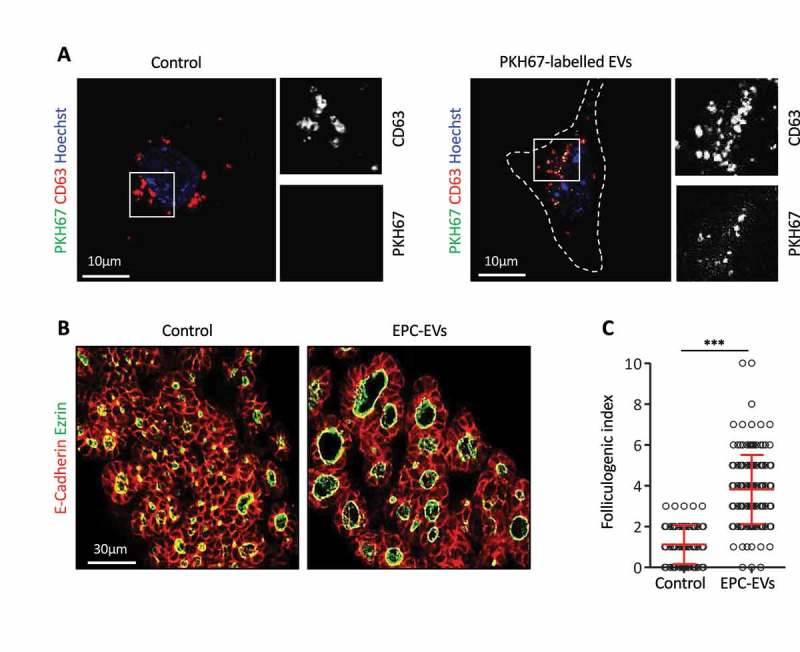
EPC-EVs are taken up by embryonic thyroid progenitors and stimulate folliculogenesis. (A) Immunofluorescence for CD63 (red) of cultured embryonic thyroid progenitors without (left panel) or after incubation with PKH67-labeled EVs (green; right panel). Cell contours are delineated by dotted lines. Note clustering around cell. High magnifications of the boxed areas at left are shown at right in single channels for CD63 and PKH signals. (B) Immunofluorescence on sections of thyroid explants cultured without (control) or with EPC-EVs for E-cadherin (red) to define epithelial cells and ezrin (green) to identify their apical pole, thus lumen contour. (C) Folliculogenic index in control- and EPC-EVs-treated explants determined by quantification of ezrin+ open follicles (***p < 0.001; *n* = 5).

We next examined whether these EVs could be incorporated by thyrocyte progenitors. To this aim, EPC-EVs were labelled with PKH67, a fluorescent lipid dye allowing imaging of EVs, then incubated with microdissected E14.5 thyroid lobes cultured on filters [16]. Despite numerous attempts (varying concentration and incubation time), we were unable to visualise fluorescent EVs in this 3D culture system of thyroid lobes due to tissue autofluorescence (see Discussion). We thus simplified the culture system, by working in 2D on adherent thyroid cells. E14.5 thyroid lobes were dissociated and the cell suspension was cultured on Ibidi**®** µ-slides coated with type IV collagen, to improve adhesion of thyrocyte progenitors. After overnight culture, most cultured cells showed weak, but non-questionable immunoreactivity for the thyroid specific marker Nkx2.1 and for epithelial cadherin (E-cadherin; data not shown), demonstrating their thyrocyte origin but suggesting dedifferentiation [25]. All cells showed punctiform labelling for CD63, most probably corresponding to endosomes or multivesicular bodies (Figure 2(A) left panel). Upon addition of EPC-EVs stained with PKH67 to the culture, exclusive colocalisation of PHK67 signal with some CD63 dots indicated that PKH67-labelled vesicles were taken up into multivesicular bodies of embryonic thyroid cells (Figure 2(A) right panel), confirming biochemical characterisation of 150k pellet (Figure 1(C)). Of note, these structures displayed a similar subcellular localisation and size as endogenous CD63^+^/PKH67^−^ structures (Figure 2(A)). We concluded that E14.5 thyrocyte progenitors could incorporate EVs produced by EPCs.

### EPC-EVs stimulate folliculogenesis

To then test whether EPC-EVs were biologically active and able to stimulate folliculogenesis, we returned to microdissected E14.5 thyroid lobes. Such explants were cultured on semi-permeable filters at the air:liquid interface [16] with or without EPC-EVs. As consistent increase in follicular lumen opening, i.e. folliculogenic activity, was previously revealed with 10× concentrated EPC-CM [4,5], preliminary experiments were conducted with 10× concentrated EPC-EVs. At this concentration, only a weak folliculogenic effect was detected after 3 days, consistent with loss of EVs during the purification procedure. However, using an additional two-fold concentration (i.e. 20× EPC-EVs), folliculogenesis was reproducibly induced (Figure 2(B)). We therefore performed all subsequent analyses with this 20X EPC-EVs concentration. Quantification revealed that EPC-EVs were able to stimulate folliculogenesis by three- to four-fold (Figure 2(C)).

**Figure 3.**
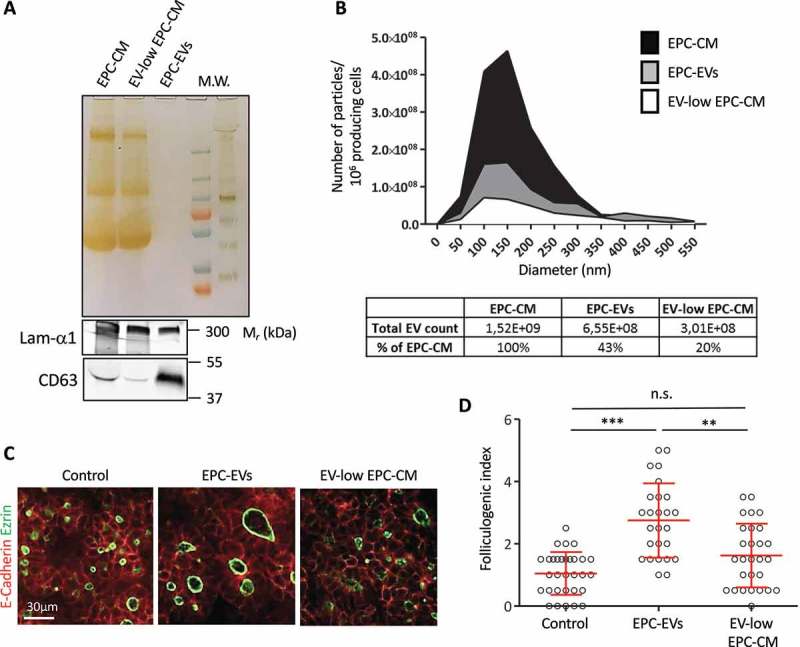
Depletion of EVs in the EPC-CM abrogates folliculogenic activity. (A) Silver staining and western blot analysis using CD63 and laminin-α1 antibodies on equal volume of 10× EPC-CM, 10× EV-low EPC-CM and 20× EPC-EVs. (B) Quantification and size distribution of particles in EPC-CM, supernatant (EV-low EPC-CM) and 150k pellet from ultracentrifuged EPC-CM (EPC-EVs), obtained by Nanoparticle Tracking analysis using a Zetaview (Particle Metrix). (C) Immunofluorescence of whole thyroid explant cultured without (Control) or with EPC-EVs (20X concentration) or EV-low EPC-CM (10× concentration). Epithelial cells (E-cadherin) are visualised in red and their apical pole (ezrin) is labelled in green. (D) Quantification of the folliculogenic effect of EPC-EVs and EV-depleted EPC-CM (***p < 0.001; ** *p* < 0.01; six explants per condition were analysed in two independent experiments).

To confirm that EVs were the bioactive factor in EPC-CM, we performed the reverse experiment and depleted EVs from the EPC-CM by ultracentrifugation of the EPC-CM (90 min, 150,000× *g*). Although ultracentrifugation did not change protein abundance in the supernatant as compared to total EPC-CM (Figure 3(A), silver staining), this leads to partial depletion of EVs, as visualised by decreased CD63 intensity (Figure 3(A), western blot). Ultracentrifuged medium was thus called EV-low EPC-CM. Conversely, despite the absence of stained proteins (silver staining), abundant CD63 signal was found in the pellet (EPC-EVs). Furthermore, particle analysis revealed that ultracentrifuged EPC-CM contained only 20% of the particles present in the starting EPC-CM, while the EPC-EVs contained at least 40% (Figure 3(B)). Finally, we tested the biological activity of the EV-low EPC-CM, at the same concentration (10×) as total EPC-CM. In line with our hypothesis, this EV-low EPC-CM had no folliculogenic activity (Figure 3(C and D)). We concluded that EVs produced by EPCs are able to promote robust expansion of the follicular lumen.

### EPC-EVs preparations contain laminins and known exosomal markers

To screen for molecules responsible for the folliculogenic activity, we next aimed to define the protein composition of EPC-EVs preparations. As a further guide in the search for a putative folliculogenic factor, we compared the 150k pellets from EPC-CM and from Human Umbilical-Vein Endothelial Cells (HUVEC)-CM, as the latter does not stimulate folliculogenesis in our thyroid lobe culture system (data not shown). EPC-EVs and HUVEC-EVs preparations were thus processed in parallel, enzymatically digested and resulting peptides identified by mass spectrometry. This analysis revealed the presence of several proteins well known to be enriched in EVs such as CD9 and syntenin-1, as well as other proteins commonly found in mass spectrometry analysis of EVs (Exocarta & Vesiclepedia). In addition, and quite interestingly, MS analysis also identified as major hit numerous peptides derived from laminin-α1 (Table 1) in EPC-EVs but not in HUVEC-EVs. Western blot indeed confirmed the presence of laminin-α1 in EPC-CM [5], and EPC-EVs (Figure 3(A)). These data suggested co-sedimentation of laminin with EVs. This extracellular matrix component has already been found to be associated with EVs (Exocarta), and to be involved in thyroid folliculogenesis [5]. As we now demonstrated that EPC-CM also contained EVs, the folliculogenic effect could be mediated by laminins, by EVs, or by their combination. To distinguish between these possibilities, we used two different approaches.

### Knockdown of laminin-α1 in EPC abrogates folliculogenic activity of the 150k pellet

**Figure 4.**
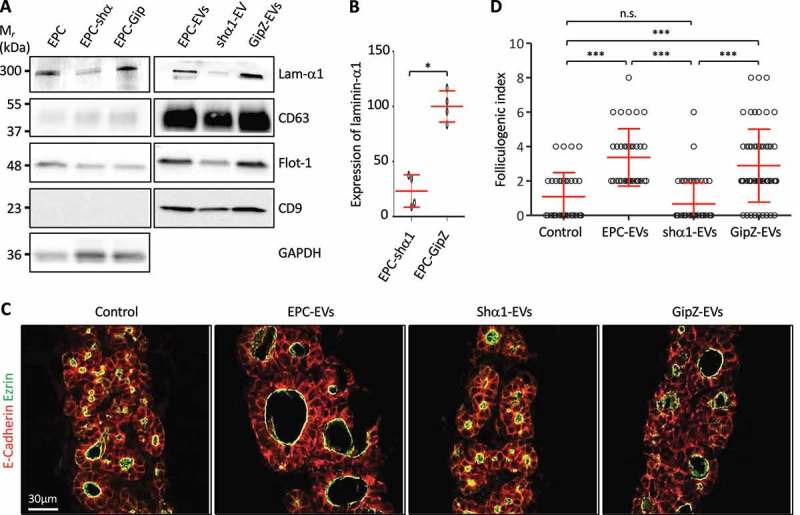
Knockdown of laminin-α1 in EPC abrogates folliculogenic activity of the 150k pellet. (A) Western blot analysis of laminin-α1, CD63, flotilin-1 and CD9 on EPC-EVs, shα1-EVs and GipZ-EVs lysates, as compared to parental cell line lysates. (B) Quantification of laminin-α1 knockdown in infected cell lysates using GAPDH as a control (*p < 0.05; *n* = 4). (C) Immunofluorescence of thyroid explant sections after culture without (control) or with EPC-EVs, shα1-EVS or GipZ-EVs as indicated. Epithelial cells (E-cadherin) are labelled in red and their apical pole (ezrin), in green. (**D)** Quantification of the folliculogenic effect of EPC-EVs, shα1-EVs and GipZ-EVs (***p < 0.001; *n* = 5).

We first decided to reduce laminin production in EPCs using a knockdown strategy with shRNA directed against laminin-α1 [5]. EVs were isolated from the CM of silenced cells and the extent of the knockdown was evaluated in the cell lysate and the 150k pellet. Western blot analysis revealed a 75% decrease of laminin-α1 in shRNA-infected EPC (EPC-shα1), as compared to cells infected with a control shRNA (EPC-GipZ; Figure 4(A) and (B)) and a similar decrease was observed in the EV preparation isolated therefrom (shα1-EVs; Figure 4(A)). However, biochemical characterisation of EVs isolated from laminin-α1 shRNA-infected EPCs also revealed a weaker signal for CD63 and flotilin-1, suggesting decreased production of total, or of a specific subpopulation of, EVs. We nevertheless tested the biological activity of shα1-EVs on thyroid lobe cultures. When shα1-EV preparation was added to E14.5 thyroid lobes, induction of folliculogenesis was abrogated (Figure 4(C) and (D)). The loss of folliculogenic activity was not due to the transfection process as EVs produced by control shRNA-infected EPC (GipZ-EVs) stimulated folliculogenesis as efficiently as control EPC-EVs (Figure 4(D)). These results indicated that laminin α1, present in the 150k pellet, is essential to promote expansion of follicles. However, since we observed a concomitant decrease in the abundance of two EVs markers in shα1-EVs (Figure 4(A)), the lack of folliculogenic activity could be attributed to the loss of a particular EV population, enriched in CD63 and flotilin-1.

### Both EPC-EVs and laminins stimulate folliculogenesis independently

**Figure 5.**
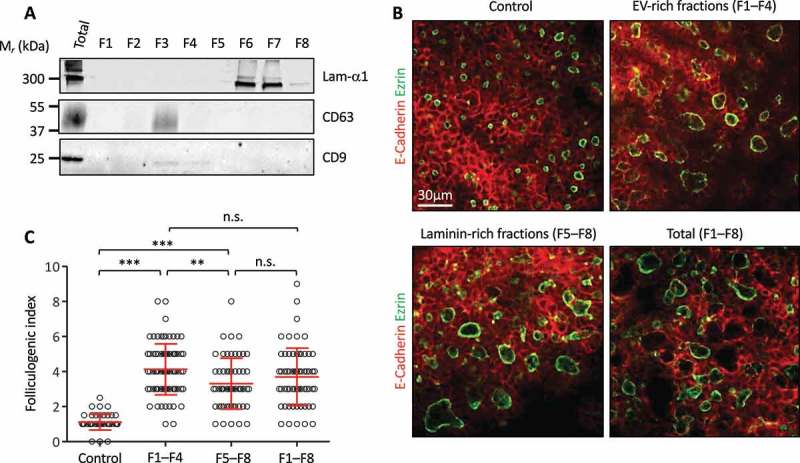
Independent effect of EPC-EVs and EPC-laminins on thyroid folliculogenesis. (A) Western blot analysis of laminin-α1, CD63 and CD9 after separation of EPC-EVs by floatation on iodixanol gradient. (B) Immunofluorescence of whole thyroid explant cultured without (control) or with pooled fractions 1 to 4 (F1–F4), 5 to 8 (F5–F8) and 1 to 8 (F1–F8). Epithelial cells (E-cadherin) are labelled in red and their apical pole (ezrin) in green. A single section is shown for each condition. (C) Quantification of the folliculogenic effect of F1–F4, F5–F8 and F1–F8 (***p < 0.001; ** *p* < 0.01; *n* = 3).

In a second approach, and in order to avoid any changes in the gene expression programme, be it in the biology of the EPC or in EV production, we decided to physically separate EVs from laminins by density-gradient centrifugation. After floatation of the 150k pellet isolated from EPC using an iodixanol density gradient, we were indeed able to achieve separation of EVs (predominantly floating in gradient fractions 2–3, as expected for vesicles) from the bulk of laminins, found predominantly in fractions 6–7 (dense, as expected for proteins), (Figure 5(A)). We pooled separately fractions 1–4 and fractions 5–8 to generate EV-rich and laminin-rich preparations, respectively. Particle analysis indeed revealed that ~60% of the initial particles were found in F1-4 vs. ~20% in F5-8 (data not shown). We then tested the capacity of the EV-rich fractions (F1–F4) and laminin-rich fractions (F5–F8) to stimulate folliculogenesis in E14.5 thyroid lobes culture. To verify that biological activity was not affected by the density gradient, we also prepared a fully reconstituted 150k pellet by mixing all fractions (F1–F8). Both EV-rich (F1–F4) and laminin-rich (F5–F8) fractions, as well as the fully reconstituted 150k pellet (F1–F8), were able to induce a robust folliculogenic response (Figure 5(B)). Furthermore, we observed a slightly stronger folliculogenic activity for the laminin-depleted EV-rich fractions as compared to EV-depleted laminin-rich fractions (Figure 5(C)). Combination of all eight fractions was not additive, indicating a saturable process (Figure 5(C)).

Altogether, these results (i) confirm that laminins can stimulate folliculogenesis on their own, (ii) indicate for the first time that laminin-depleted EPC-EVs exhibit pro-folliculogenic potential and (iii) suggest that laminin impacts on the production of EVs by endothelial cells and/or their effect in thyrocytes.

## Discussion

In the present study, we report that embryonic EPCs produce EVs that can be internalised by thyroid progenitor cells, and are able to stimulate the expansion of follicular lumen, i.e. folliculogenesis, in thyroid lobes cultured *ex vivo*.

Characterisation of the 150k pellet, obtained from the culture medium conditioned by EPCs, revealed the presence of round vesicles with a mean size between 100 and 150 nm, equilibrating in iodixanol gradients at a density around 1.12 and enriched in bonafide EV markers such as CD63, CD9 and flotilin-1, without detectable ER marker, calnexin. These three characteristics support the claim that the 150k pellet contains EVs [11,24]. It is widely accepted that the differential ultracentrifugation protocol, used in our study and by most EVs research groups [26–28], does not allow optimal separation of exosomes and MVs. Indeed, small-sized MVs are likely present in the 150k pellet [29,30]. We nevertheless claim that the folliculogenic effect reported in this study is mediated by exosomes rather than by MVs. Indeed, we found that the 20k pellet, reported to be enriched in MVs, had no detectable effect on folliculogenesis (data not shown). Considering the heterogeneity of vesicles and the absence of definitive specific markers for the different EV subpopulations [19,31], we did not further investigate this aspect and used the term EVs to refer to the vesicles produced by EPCs and present in the 150k pellet.

We were unable to convincingly visualise incorporation of EPC-EVs in whole 3D thyroid lobes, probably due to the thickness of the samples associated with high auto-fluorescence of the 3D tissue pieces. However, we demonstrated that PKH-labelled-EVs were readily incorporated within isolated embryonic thyroid cells in culture. Although most cells had only a weak staining for E-cadherin and the thyroid transcription factor (Nkx2.1), we observed that fluorescent EVs were captured by thyroid progenitors. The loss of E-cadherin and Nkx2.1 is likely due to dedifferentiation initiated upon tissue dissociation and culture [25]. Another study reported that primary cultures of adult thyroid cells require a complex supplementation of culture medium with growth hormones to maintain the differentiated state [32]. Based on the internalisation of EVs in dissociated thyroid progenitor cells, we postulate that stimulation of folliculogenesis in 3D thyroid explants could be due to the entry of EVs, and delivery of their cargo, into the thyroid cells. However, we cannot exclude the possibility that vesicles act on thyroid cells by binding to cell surface receptors and triggering cellular changes, without entering into the target cell.

We observed that addition of the 150k pellet on embryonic thyroid lobes in culture induces a robust folliculogenic response. Mass spectrometry analysis of the 150k pellet further revealed, besides canonical EV protein markers, the presence of abundant laminin peptides. We do not think that laminins are carried within EVs. Indeed, laminins being secreted proteins could not be incorporated within EVs during MV budding or intraluminal vesicle formation in multivesicular endosomes. Laminins must either co-sediment with EVs or interact biochemically with EVs. Indeed, when EPC-EVs were treated with trypsin, we observed a loss of the laminin α1 signal but not of CD63 (data not shown). Interestingly, we previously reported that follicular lumen expansion is promoted by the presence of trimeric laminin-α1, β1, γ1 (also known as laminin-111) in the CM produced by EPC, and that CM can rescue folliculogenesis in two different knockout models causing defective follicle formation [4,5]. Demonstration in the present report that CM contains EVs now revealed that folliculogenic effects of conditioned media could be attributed to EVs. To better define the respective role of trimeric laminins and EVs, two types of experiments were performed: (i) knockdown of laminin-α1 and (ii) physical separation of EVs from laminins on a density gradient. In the first approach, EVs isolated from medium produced by EPC knocked down for laminin-α1 failed to induce folliculogenesis, suggesting the possibility that trimeric laminin (α1 β1 γ1) could be the folliculogenic factor, or that laminin was essential for the folliculogenic response. Surprisingly, biochemical characterisation of EVs produced by EPC knocked down for laminin-α1 revealed a concomitant decrease in EV markers as compared to control EPC, suggesting impairment of the total production of EVs, and/or orientation into a distinct EV subtype upon laminin-α1 silencing. In this view, suppression of the folliculogenic effect after laminin-α1 knockdown would be related to altered production of bioactive EVs. A role of laminin-111 on EVs biogenesis or release has not yet been shown. However, it has been demonstrated that other extracellular matrix component, i.e. heparan sulphate proteoglycans, influences EVs production by triggering intracellular signalling [33]. In the second approach, we attempted to physically separate EVs from laminins by iodixanol gradient. Surprisingly, both laminin-rich fractions (F5–F8), and EV-rich fractions (F1–F4) were able to stimulate the expansion of thyroid follicles in *ex vivo* culture of embryonic thyroid lobes. However, these data do not rule out the possibilities that laminin by itself promoted the folliculogenic activity of endogenous EV, produced by cultured lobes, and that residual content of the sticky protein, laminin, in EV-rich fractions (F1–F4) contributed to their effective targeting for signalling into thyrocytes. Hence, our previously reported rescues of defective folliculogenesis in *Vegfa* KO [4] and *Smad1/Smad5* double KO [5] using medium conditioned by EPC is probably due to the concerted action of EVs and of laminins released into the CM. To further test this hypothesis *in vivo* and to identify the key folliculogenic factor transported via EVs from endothelial cells to thyrocyte progenitors are therefore important future challenges. Mass spectrometry analysis of EV-rich fractions (F1–F4) might also reveal if laminins are completely depleted, and whether other candidate proteins might be responsible for the folliculogenic activity. To extend our observations to a physiological situation and demonstrate a role for EVs *in vivo* during thyroid development, we initiated experiments to isolate and characterise EVs from dissociated embryonic thyroid lobes. However, despite the presence of CD63^+^ vesicles in the 150k pellet, the number of animals required to isolate sufficient amount of EVs for biochemical analysis and functional experiment was prohibitory. Nevertheless, we hope that improvement in EV isolation and analysis methods, including omics, will help to address these questions.

In conclusion, we here demonstrated that embryonic EPCs produce EVs with exosomal characteristics, able to stimulate embryonic thyroid folliculogenesis in an *ex vivo* culture system of thyroid lobes. Our data also suggest that the production of folliculogenic EVs by EPCs depends on laminin-α1 expression and raise the possibility that laminin can promote the effect of endogenous EVs.

## References

[1] Fagman H, Andersson L, Nilsson M. The developing mouse thyroid: embryonic vessel contacts and parenchymal growth pattern during specification, budding, migration, and lobulation. Dev Dyn. 2006 2;235(2):444–12. 16331648 10.1002/dvdy.20653

[2] Colin IM, Denef JF, Lengelé B, et al Recent insights into the cell biology of thyroid angiofollicular units. Endocr Rev. 2013 4;34(2):209–238. 23349248 10.1210/er.2012-1015 PMC3610675

[3] Nilsson M, Fagman H Development of the thyroid gland. Development. 2017 6 15;144(12):2123–2140. 28634271 10.1242/dev.145615

[4] Hick AC, Delmarcelle AS, Bouquet M, et al Reciprocal epithelial: endothelialparacrine interactions during thyroid development govern follicular organization and C-cells differentiation. Dev Biol. 2013 9 1;381(1):227–240. 23707896 10.1016/j.ydbio.2013.04.022

[5] Villacorte M, Delmarcelle AS, Lernoux M, et al Thyroid follicle development requires Smad1/5- and endothelial cell-dependent basement membrane assembly. Development. 2016 6 1;143(11):1958–1970. 27068110 10.1242/dev.134171 PMC4920163

[6] Raposo G, Stoorvogel W Extracellular vesicles: exosomes, microvesicles, and friends. J Cell Biol. 2013 2 18;200(4):373–383. 23420871 10.1083/jcb.201211138 PMC3575529

[7] Pan BT, Johnstone RM Fate of the transferrin receptor during maturation of sheep reticulocytes in vitro: selective externalization of the receptor. Cell. 1983 7;33(3):967–978. 6307529 10.1016/0092-8674(83)90040-5

[8] Harding C, Heuser J, Stahl P Receptor-mediated endocytosis of transferrin and recycling of the transferrin receptor in rat reticulocytes. J Cell Biol. 1983 8;97(2):329–339. 6309857 10.1083/jcb.97.2.329 PMC2112509

[9] Raposo G, Nijman HW, Stoorvogel W, et al B lymphocytes secrete antigen-presenting vesicles. J Exp Med. 1996 3 1;183(3):1161–1172. 8642258 10.1084/jem.183.3.1161 PMC2192324

[10] Valadi H, Ekström K, Bossios A, et al Exosome-mediated transfer of mRNAs and microRNAs is a novel mechanism of genetic exchange between cells. Nat Cell Biol. 2007 6;9(6):654–659. 17486113 10.1038/ncb1596

[11] Yáñez-Mó M, Siljander PR, Andreu Z, et al Biological properties of extracellular vesicles and their physiological functions. J Extracell Vesicles. 2015 5;14(4):27066. 10.3402/jev.v4.27066 PMC4433489 25979354

[12] Meehan B, Rak J, Di Vizio D Oncosomes - large and small: what are they, where they came from? J Extracell Vesicles. 2016 9;27(5):33109. 10.3402/jev.v5.33109 PMC5040817 27680302

[13] Tkach M, Théry C Communication by extracellular vesicles: where we are and where we need to go. Cell. 2016 3 10;164(6):1226–1232. 26967288 10.1016/j.cell.2016.01.043

[14] Hayashi T, Lombaert IM, Hauser BR, et al Exosomal MicroRNA transport from salivary mesenchyme regulates epithelial progenitor expansion during organogenesis. Dev Cell. 2017 1 9;40(1):95–103. 28041903 10.1016/j.devcel.2016.12.001 PMC6720111

[15] Consortium EV-TRACK, Van Deun J, Mestdagh P, et al EV-TRACK: transparent reporting and centralizing knowledge in extracellular vesicle research. Nat Methods. 2017 2 28;14(3):228–232. 28245209 10.1038/nmeth.4185

[16] Delmarcelle AS, Villacorte M, Hick AC, et al An ex vivo culture system to study thyroid development. J Vis Exp. 2014;6:88. 10.3791/51641 PMC4186730 24961920

[17] Pierreux CE, Poll AV, Kemp CR, et al The transcription factor hepatocyte nuclear factor-6 controls the development of pancreatic ducts in the mouse. Gastroenterology. 2006 2;130(2):532–541. 16472605 10.1053/j.gastro.2005.12.005

[18] Hatzopoulos AK, Folkman J, Vasile E, et al Isolation and characterization of endothelial progenitor cells from mouse embryos. Development. 1998 4;125(8):1457–1468. 9502726 10.1242/dev.125.8.1457

[19] Kowal J, Arras G, Colombo M, et al Proteomic comparison defines novel markers to characterize heterogeneous populations of extracellular vesicle subtypes. Proc Natl Acad Sci USA. 2016 2 23;113(8):E968–77. 26858453 10.1073/pnas.1521230113 PMC4776515

[20] Cominelli A, Gaide Chevronnay HP, Lemoine P, et al Matrix metalloproteinase-27 is expressed in CD163+/CD206+ M2 macrophages in the cycling human endometrium and in superficial endometriotic lesions. Mol Hum Reprod. 2014 8;20(8):767–775. 24810263 10.1093/molehr/gau034

[21] Tyteca D, D’Auria L, Van Der Smissen P, et al Three unrelated sphingomyelin analogs spontaneously cluster into plasma membrane micrometric domains. Biochim Biophys Acta. 2010 5;1798(5):909–927. 20123084 10.1016/j.bbamem.2010.01.021

[22] Théry C, Amigorena S, Raposo G, et al Isolation and characterization of exosomes from cell culture supernatants and biological fluids. Curr Protoc Cell Biol. 2006 4; Chapter 3: Unit3.22:1-29. 10.1002/0471143030.cb0322s30 18228490

[23] Witwer KW, Buzás EI, Bemis LT, et al Standardization of sample collection, isolation and analysis methods in extracellular vesicle research. J Extracell Vesicles. 2013 5 27;2. 10.3402/jev.v2i0.20360 PMC3760646 24009894

[24] Lötvall J, Hill AF, Hochberg F, et al Minimal experimental requirements for definition of extracellular vesicles and their functions: a position statement from the international society for extracellular vesicles. J Extracell Vesicles. 2014 12;22(3):26913. 10.3402/jev.v3.26913 PMC4275645 25536934

[25] Suzuki K, Mitsutake N, Saenko V, et al Dedifferentiation of human primary thyrocytes into multilineage progenitor cells without gene introduction. PLoS One. 2011 4 27;6(4):e19354. 21556376 10.1371/journal.pone.0019354 PMC3083435

[26] Lässer C, Eldh M, Lötvall J Isolation and characterization of RNA-containing exosomes. J Vis Exp. 2012 1;9(59):e3037. 10.3791/3037 PMC3369768 22257828

[27] Lobb RJ, Becker M, Wen SW, et al Optimized exosome isolation protocol for cell culture supernatant and human plasma. J Extracell Vesicles. 2015 7;17(4):27031. 10.3402/jev.v4.27031 PMC4507751 26194179

[28] Gardiner C, Di Vizio D, Sahoo S, et al Techniques used for the isolation and characterization of extracellular vesicles: results of a worldwide survey. J Extracell Vesicles. 2016 10;31(5):32945. 10.3402/jev.v5.32945 PMC5090131 27802845

[29] Bobrie A, Krumeich S, Reyal F, et al Rab27a supports exosome-dependent and -independent mechanisms that modify the tumor microenvironment and can promote tumor progression. Cancer Res. 2012 10 1;72(19):4920–4930. 22865453 10.1158/0008-5472.CAN-12-0925

[30] Zabeo D, Cvjetkovic A, Lässer C, et al Exosomes purified from a single cell type have diverse morphology. J Extracell Vesicles. 2017 6 20;6(1):1329476. 28717422 10.1080/20013078.2017.1329476 PMC5505001

[31] Willms E, Johansson HJ, Mäger I, et al Cells release subpopulations of exosomes with distinct molecular and biological properties. Sci Rep. 2016 3;2(6):22519. 10.1038/srep22519 PMC4773763 26931825

[32] Barington M, Brorson MM, Hofman-Bang J, et al Ghrelin-mediated inhibition of the TSH-stimulated function of differentiated human thyrocytes ex vivo. PLoS One. 2017 9 20;12(9):e0184992. 28931076 10.1371/journal.pone.0184992 PMC5607171

[33] Roucourt B, Meeussen S, Bao J, et al Heparanase activates the syndecan-syntenin-ALIX exosome pathway. Cell Res. 2015 4;25(4):412–428. 25732677 10.1038/cr.2015.29 PMC4387558

